# DOES COGNITIVE MISPERCEPTION AFFECT THE TIMING OF COMPLETING ADVANCE DIRECTIVES?

**DOI:** 10.1093/geroni/igae098.2385

**Published:** 2024-12-31

**Authors:** Yifan Lou, Zhuoer Lin, Xi Chen

**Affiliations:** Virginia Commonwealth University, New Haven, Virginia, United States; University of Illinois Chicago, Chicago, Illinois, United States; Yale University, New Haven, Connecticut, United States

## Abstract

While there has been extensive research on improving advance directives (AD) completion rates, less attention has been paid to the early engagement of AD. Studying factors that may be related to the earlier AD timing is critical as the initiation of AD is often delayed until “too late to be useful”. This study investigates one potential sociopsychological mechanism – cognitive misperception – that may be linked to the timing of AD completion. Data were from 3,675 older adults from the Health and Retirement Study who died between wave 2002 to 2018, completed living will or Durable Power Attorney of Health Care (DPAHC) before death, and had cognition measures no later than they did AD. Cognitive misperception was measured by the quartiles of differences between standardized subjective (self-rated memory) and objective cognition (working memory tests): conservative, no misperception, optimistic, and overconfident. Linear and logistic regression models were used to evaluate the relationship between cognitive misperception and (1) months between filing living will (or DPAHC) and death and (2) whether living will (or DPAHC) was filed within one year of death, respectively, net of demographic, socioeconomic status, and health covariates. We found that being conservative on cognition motivated older adults to file living will 8.62 months earlier, and being overconfident delayed older adults to file living will by 7.25 months. Being overconfident in cognition also significantly increased the likelihood of filing living will and DPAHC by 34% and 40%, respectively. Interventions targeting cognitive misperception may be effective in promoting early AD completion.

